# Rush Medical College

**Published:** 1874-11

**Authors:** Walter Hay


***Rush Medical College.*** Clinic for Diseases of Nervous System, by Dr. Walter Hay. Reported by Henry W. Harrington.

*Case of Hemicrania.*

Mrs. K——, aged forty-five. Housewife. German. Patient is a large, well nourished woman; general health good; appetite good; bowels regular. States that when a little girl, she received a violent blow upon the right temple, and supposes this to have been the cause of her trouble. She recovered entirely from the immediate effects of the injury, but for about thirty years has been subject to periodical attacks of pain in the right temple, recurring at intervals of about a week, sometimes less frequently. These attacks are ushered in by a feeling of chilliness and lassitude; also by excessive yawning; soon the pain begins, mild at first, but growing more and more severe until it amounts to perfect agony. After a time she suffers from nausea, and vomits freely, after which the pain usually begins to subside, and gradually passes away; the attacks lasting from twenty-four to thirty-six hours. The pain is always confined to one side of the head, to the supra orbital and temporal regions. Ordered—R. Sodii bromid. grs. xxx, three times daily, to be continued until bromism is induced, after which, to diminish the amount, taking grs. xxx at bed-time alone, if no more can be borne; also, that her diet be mild and easily digestible, especially at supper.

*Remarks.* This, gentlemen, is a typical case of hemicrania, or migraine; neuralgia of a portion of the fifth pair of nerves. The blow to which she refers as the cause of the disease, is such, only in a restricted sense. There was present, no doubt, a tendency to disorder of the nervous system; and then the disease was developed at a time in her life when her system was undergoing those developmental changes peculiar to woman; changes which rendered her very susceptible to this disorder. The disease is due to pathological changes in the cells of the fifth pair of nerves; just what these changes are, or how brought about, is not known. The nausea and vomiting which nearly always occur, have led some to suppose that these constitute the primary disorder, hence the name, "sick headache." They do not, but are the result of thepa in in the head, and are reflex in character. I prescribe the bromide of sodium to allay the nervous excitability and susceptibility to these attacks of pain. I prefer it to the potassium salt, because it is more easily absorbed and assimilated, and more certain in its action.

*Two Cases of Organic Infantile Paralysis.*

CASE 1. Alice D——, aged seven years. The mother states that the child was apparently healthy when born, and was well until the age of three years, when she had an attack of small-pox; was sick one month; for one year afterwards she was unable to walk, and when she began walking, the mother noticed that she did not walk well; the right leg seemed weak, and the toes dragged. After a time she noticed that the right leg and arm were smaller than the left. Patient sleeps well; appetite good. She is usually good tempered, but often has long spells of crying; she seems to understand what is said to her, but does not remember anything. On examination, find the right arm and leg much atrophied, the extensors more so than the flexors, so that the hand drops, and the toes catch in walking; right leg measures one inch less in circumference than the left. Patient is continually rolling her eyes about, and laughing in an idiotic manner; is constantly in motion, throwing her arms and legs about irregularly; pupils dilated, and only slightly sensitive to light. She was ordered—R. Strychniæ sulph. gr. $\frac{1}{60}$, ferri phosphatis gr. j, acid phosphoric dil. gtts. viij., three times daily.

CASE 2. Amelia W——, aged eight. The mother states that the child was well until she was nineteen months old, at which time she had "brain fever," which was immediately followed by her present trouble. Previous to her sickness she walked without trouble, but afterwards the toes of the left foot dragged in walking, and the left arm also became weak; she could not hold up the hand, nor extend it upon the wrist. She has continued in the same condition ever since. Appetite good; bowels regular; sleeps well. On examination, find left arm and leg much smaller and shorter than the right, corresponding in size to those of a child about two years of age. There is a greater degree of atrophy of the extensors than of the flexors, giving an appearance of contraction of the flexors, so that neither the arm nor the leg can be perfectly extended voluntarily; for the same reason the heel is elevated; left arm and leg very weak. Ordered—R. Strychniæ sulph. gr. $\frac{1}{50}$, ferri pyrophosphatis gr. j, three times daily; also—R. Olei morrhuæ dr. ss, three times daily; good diet and exercise.

*Remarks.* In these cases you recognize a disease called organic infantile paralysis. It differs materially from the paralysis of adults, in which the essential characteristic is loss of motility without at first atrophy of muscular tissue. In this disorder, there is arrest of development, due to atrophy of those cells in the nerve-centers which preside over nutrition, and the loss of motor power is due to this loss or lack of muscular tissue. In addition to this, we have, in the first case, arrest of development in the hemispheres, indicated by the lack of intelligence.

The indications to be fulfilled in the treatment, are: to increase the nutritive power of nerve-cells, also to increase general nutrition. To accomplish this, we order good diet; tonics, such as iron and strychnia; and those elements which will supply an existing deficiency in nerve-tissue, a deficiency always existing in nervous disease, indicated by the largely increased amount of phosphatic salts in the urine, and showing a waste of nerve-tissue which exceeds repair. Hence, we prescribe phosphorous in some form, on purely physiological principles. We cannot promise a cure, neither will the existing deformity disappear, but further progress of the disease may be arrested.

August 8. Dr. HAY.

*Syphilitic Neuralgia.*

A woman was presented who complained of pain radiating from the spinal centre round the body along the intercostal nerves. There was spinal tenderness, great sensitiveness to noise and light, pain in the head, and impaired hearing, signs of periostitis of the clavicle and other long bones. Ordered a fly blister, three inches by twelve, to the back, and five-grain doses of the potassium iodide. It is necessary to ascertain the cause of neuralgia; whether it be due to malaria, or nervous exhaustion, or syphilis. The fly-blister, by its reflex action on the vaso-motor nerves, irritates, and produces contraction of the internal blood vessels, lessens the exosmosis and the calibre of the vessels. The heart's action will be increased, and send along the blood faster, whereby absorption into the vessels of effete matter will be increased, and, at the same time, the quick distribution of the iodide she is now taking will be facilitated.

